# Effects of Fibroid Size and Hyaluronan-Enriched Transfer Medium on Clinical Pregnancy and Live Birth: A Retrospective Study

**DOI:** 10.3390/jcm15187176

**Published:** 2026-09-15

**Authors:** Zercan Kalı, Emrah Zayman, Nihal Mavral

**Affiliations:** 1 Department of Obstetrics and Gynecology, Private Gözde Hospital, 44100 Malatya, Türkiye; 2 Department of Histology and Embryology, Faculty of Medicine, Malatya Turgut Özal University, 44210 Malatya, Türkiye; 3 Department of Obstetrics and Gynecology, Faculty of Medicine, Malatya Turgut Özal University, 44210 Malatya, Türkiye

**Keywords:** EmbryoGlue, hyaluronan, intramural fibroid, frozen embryo transfer, in vitro fertilization, live birth

## Abstract

**Objective:** Non-cavity-distorting intramural fibroids have been linked to poorer reproductive outcomes in assisted reproduction, yet strategies to counteract this effect remain underexplored. This study examined whether a hyaluronan-enriched transfer medium (EmbryoGlue^®^) is associated with clinical pregnancy and live birth in women with such fibroids undergoing frozen-thawed single blastocyst transfer, and whether any such association depends on fibroid size. **Methods:** We conducted a single-center retrospective cohort analysis of 515 women, aged 20–37 years, carrying the International Federation of Gynecology and Obstetrics (FIGO) type 3–5 non-cavity-distorting intramural fibroids, who underwent frozen-thawed single blastocyst transfer between January 2021 and December 2025. Of these, 232 received a hyaluronan-enriched transfer medium and 283 received a standard medium. Clinical pregnancy served as the primary endpoint; positive pregnancy test, live birth, and early pregnancy loss were secondary endpoints. Multivariable logistic regression was used to identify independent predictors of clinical pregnancy and live birth and to test for an interaction between EmbryoGlue use and fibroid size. **Results:** Women who received EmbryoGlue had higher rates of positive pregnancy test (50.0% vs. 32.9%; *p* < 0.001), clinical pregnancy (40.5% vs. 26.9%; *p* = 0.001), and live birth (30.2% vs. 19.1%; *p* = 0.003) than those in the control group. After adjustment, and taking a fibroid size of 3 cm as the reference point, EmbryoGlue use remained associated with greater odds of clinical pregnancy (adjusted odds ratio [aOR] 2.05, 95% CI 1.20–3.50; *p* = 0.008). A significant interaction between EmbryoGlue use and fibroid size emerged for both clinical pregnancy (aOR 1.74, 95% CI 1.13–2.68; *p* = 0.011) and live birth (aOR 1.68, 95% CI 1.06–2.67; *p* = 0.028): the association between EmbryoGlue and reproductive outcomes was not uniform but depended on fibroid size, with the widest gap between groups seen among women with fibroids exceeding 4 cm. **Conclusions:** Among women with non-cavity-distorting intramural fibroids undergoing frozen-thawed single blastocyst transfer, EmbryoGlue use was linked to more favorable reproductive outcomes overall, an association that varied by fibroid size and was most pronounced above 4 cm. These findings warrant confirmation through prospective randomized trials before influencing clinical practice.

## 1. Introduction

Whether non-cavity-distorting intramural fibroids compromise the success of assisted reproductive technology (ART) remains a matter of ongoing debate, although several recent meta-analyses point toward a detrimental influence on clinical pregnancy and live birth rates [1,2,3]. In a pooled analysis of 5029 women, Rikhraj and colleagues reported that live birth was roughly 44% less likely in the presence of these fibroids [1]. Sunkara and colleagues similarly documented worse outcomes even when the uterine cavity itself was not distorted [2], while Erden and colleagues described reduced clinical pregnancy and live birth rates for fibroids up to 6 cm in size [3]. Most of the underlying evidence, however, comes from retrospective cohorts, leaving open questions about how fibroid size and specific treatment approaches interact to shape in vitro fertilization (IVF) success.

Intramural fibroids are among the most common uterine pathologies encountered in women of reproductive age, and their management in the context of infertility remains a persistent clinical dilemma. Surgical removal carries its own risks, including bleeding, adhesion formation, and a potential reduction in ovarian reserve, while a conservative approach leaves the fibroid’s possible adverse influence on implantation unaddressed [1,2,3]. This tension has motivated growing interest in non-surgical strategies that might mitigate the impact of fibroids on IVF outcomes without exposing patients to operative risk.

Successful implantation depends on a combination of factors, including embryo quality, the receptivity of the endometrium, and the molecular dialogue that takes place between the two [4,5]. Various adjuncts have been explored to optimize this environment, ranging from modifications of the transfer medium itself to endometrial injection of embryo culture supernatant [6]. Among these, supplementing the transfer medium with hyaluronan—a glycosaminoglycan found naturally in cervical mucus, follicular fluid, and endometrial tissue, and believed to promote implantation through CD44-receptor interactions on both the embryo and the endometrium [5,7]—has received particular attention in the literature. Several clinical trials and meta-analyses evaluating hyaluronan-enriched media report modest gains in clinical pregnancy and live birth rates in certain patient groups, lending support to the broader hypothesis that such media can reinforce embryo–endometrium interaction [7].

EmbryoGlue^®^, a transfer medium enriched with hyaluronan, is designed to facilitate closer contact and adhesion between the embryo and the endometrial surface. Evidence on how well it performs specifically in women with intramural fibroids is scarce, and no study to date has adequately examined whether its performance depends on fibroid size. Because non-cavity-distorting fibroids are thought to impair endometrial receptivity and implantation through mechanical or functional pathways [1,2,3], we reasoned that a hyaluronan-enriched medium might offer a proportionally greater benefit in this specific population, and that this benefit might become more evident as fibroid size increases. Accordingly, the present study set out to examine the relationship between EmbryoGlue use and clinical pregnancy and live birth among women with non-cavity-distorting intramural fibroids undergoing frozen-thawed single blastocyst transfer, and to test whether this relationship is modified by fibroid size (EmbryoGlue × fibroid-size interaction).

## 2. Materials and Methods

### 2.1. Study Design

This retrospective single-center cohort study was based on a review of the clinical records of women who had undergone IVF/intracytoplasmic sperm injection (ICSI) treatment at the Private Malatya Gözde Academy IVF Center, Türkiye, between January 2021 and December 2025. A total of 515 women with non-cavity-distorting intramural fibroids who met the study eligibility criteria were included. Clinical, embryological, and reproductive outcome data were retrieved from the institutional electronic medical records. Throughout the study period, all ovarian stimulation protocols, embryo culture conditions, embryo grading procedures, vitrification–warming protocols, and frozen-thawed single blastocyst transfer procedures were performed according to standardized institutional protocols.

The study was reported in accordance with the Strengthening the Reporting of Observational Studies in Epidemiology (STROBE) statement.

### 2.2. Ethical Approval

The study was conducted in accordance with the principles of the Declaration of Helsinki. The study protocol was submitted to the Ethics Committee of Malatya Turgut Özal University on 1 November 2025 (Application No. 2025/415) and was approved by the Ethics Committee on 16 February 2026. Due to the retrospective nature of the study and the use of anonymized clinical data, the requirement for written informed consent was waived by the Ethics Committee.

### 2.3. Patient Selection

Patients with non-cavity-distorting intramural fibroids who underwent frozen-thawed single blastocyst transfer were included in the study. Patients were divided into two groups according to the transfer medium used: the EmbryoGlue group and the standard transfer medium (control) group.

Inclusion criteria:

- Age between 20 and 37 years;
- Presence of a non-cavity-distorting intramural fibroid confirmed by Ttransvaginal ultrasonography (TVUS);
- Frozen-thawed single blastocyst transfer performed on day 5 or 6;
- Availability of follow-up data on clinical pregnancy and live birth outcomes.

The upper age limit of 37 years was set to minimize the confounding effect of advanced maternal age—associated with increased embryo aneuploidy, reduced implantation potential, and increased risk of pregnancy loss [8]—on the study outcomes.

Exclusion criteria:

- Fresh embryo transfer cycles;
- Double embryo transfers;
- Cleavage-stage embryo transfers;
- Submucosal fibroids or fibroids distorting the uterine cavity;
- Endometrial polyp;
- Intrauterine adhesion;
- Endometrial thickness <7 Mm;
- Congenital uterine anomaly;
- Patients with aissing clinical data.

### 2.4. Assessment of Intramural Fibroids

All fibroids were assessed by experienced gynecologists using TVUS. Fibroids were classified according to the International Federation of Gynecology and Obstetrics (FIGO) leiomyoma classification system, and only non-cavity-distorting intramural fibroids (FIGO type 3, 4, and 5) were included in the study. Submucosal fibroids (FIGO type 0, 1, 2) and fibroids distorting the uterine cavity were excluded. Cavity involvement was confirmed by TVUS in all patients. Saline infusion sonography (SIS) was additionally performed when the fibroid was located close to the endometrial–myometrial interface, when possible endometrial contour irregularity or compression was observed, or when TVUS findings were inconclusive regarding cavity distortion, with particular attention to confirming cavity integrity in larger (≥4 cm) fibroids located close to the endometrium. Using these criteria, SIS was performed in 83/515 (16.1%) patients—39/232 (16.8%) in the EmbryoGlue group and 44/283 (15.5%) in the control group (*p* = 0.698)—with no significant difference in SIS utilization between groups. No patient in whom SIS demonstrated cavity distortion was included in the final cohort.

In patients with multiple fibroids, the maximum diameter of the largest fibroid was used in the analyses. Fibroid size was recorded as the largest diameter measured in three orthogonal planes, and fibroids were classified by size as <2 cm, 2–4 cm, and >4 cm. Fibroid size was also evaluated as a continuous variable (per cm) in multivariable analyses.

### 2.5. Ovarian Stimulation and Embryo Development

Controlled ovarian stimulation was performed using a GnRH antagonist protocol in all patients. Stimulation was initiated with individualized doses of recombinant follicle-stimulating hormone (FSH) (Gonal-F^®^, Merck Serono, Geneva, Switzerland) and/or human menopausal gonadotropin (Menopur^®^, Ferring, Kiel, Germany), based on the patient’s age, anti-Müllerian hormone (AMH) level, and antral follicle count (AFC). A GnRH antagonist (Cetrotide^®^, Merck Serono, Geneva, Switzerland; 0.25 mg/day) was added once the leading follicle reached 12–14 mm in diameter to prevent premature luteinization. Final oocyte maturation was triggered with recombinant hCG (Ovitrelle^®^, Merck Serono, Geneva, Switzerland; 250 µg) once the majority of follicles reached 18–20 mm in diameter, and oocyte retrieval was performed under transvaginal ultrasound guidance 34–36 h later.

Fertilization was achieved by ICSI in all cases. Embryos were cultured to the blastocyst stage in a MIRI^®^ multiroom benchtop incubator (Esco Medical, Singapore) under low-oxygen conditions (37 °C, 6% CO_2_, and 5% O_2_); time-lapse imaging was not used during the study period. Embryo selection was based solely on morphological assessment.

### 2.6. Embryo Grading

Blastocysts were graded according to the Gardner and Schoolcraft classification system. Good-quality blastocysts were defined as AA, AB, and BA. Good-quality blastocysts were cryopreserved by vitrification.

### 2.7. Endometrial Preparation and Embryo Transfer

Embryo transfers were performed in hormone replacement therapy (HRT) cycles. Endometrial preparation was achieved with oral estradiol valerate (Estrofem^®^, Novo Nordisk, Bagsværd, Denmark; 6 mg/day) started on day 2–3 of the menstrual cycle. Once an endometrial thickness of ≥7 mm and an appropriate trilaminar pattern were achieved, luteal phase support was initiated with vaginal progesterone (Crinone^®^ 8% gel, Merck KGaA, Darmstadt, Germany; 90 mg/day). Frozen-thawed single blastocyst transfer was performed on the sixth day after the initiation of progesterone.

In the EmbryoGlue group, blastocysts to be transferred were incubated in a hyaluronan-rich embryo transfer medium (EmbryoGlue^®^, Vitrolife, Gothenburg, Sweden) for approximately 15–30 min according to the manufacturer’s recommendations, and were subsequently loaded into the transfer catheter in the same medium. In the control group, blastocysts were incubated in a standard embryo transfer medium (G-2 Plus™, Vitrolife, Gothenburg, Sweden) prior to transfer and were subsequently transferred using the same embryo transfer protocol. All transfers were performed under ultrasound guidance using a soft embryo transfer catheter (Cook Medical, Bloomington, IN, USA).

At our center, EmbryoGlue was included as part of the standard IVF treatment package at no additional cost and was recommended as a routine adjunctive option to all patients meeting the eligibility criteria throughout the study period. The recommendation was independent of patient demographics, infertility etiology, ovarian reserve, embryo quality, fibroid size, or anticipated prognosis. Following standardized counseling, the decision to use EmbryoGlue was based solely on patient preference. Accordingly, treatment allocation was not driven by clinician-selected prognostic factors, and financial considerations did not influence the decision, as EmbryoGlue was provided without extra charge. Prior failed embryo transfer history was compared between groups and did not differ significantly (Table 1).

### 2.8. Follow-Up and Outcome Measures

Biochemical pregnancy was assessed by measuring serum β-hCG levels 12–14 days after embryo transfer. Patients with a positive test were re-evaluated by TVUS 2–4 weeks later.

The primary outcome of the study was the clinical pregnancy rate. Secondary outcomes were the live birth rate, positive pregnancy test rate, and early pregnancy loss rate.

Outcome definitions:

- Positive Pregnancy Test: Positive Serum B-Hcg on Post-Transfer Day 12–14;
- Clinical Pregnancy: Visualization of an Intrauterine Gestational Sac on TVUS;
- Live Birth: Delivery of A Live Infant After 22 Weeks of Gestation;
- Early pregnancy loss: pregnancy loss occurring before 20 weeks of gestation following a clinical pregnancy;
- All pregnancies were followed until a definitive outcome (live birth or pregnancy loss) was reached, with complete outcome data available for all patients as of the final follow-up date of June 2026. No pregnancy had an unresolved or missing outcome at the time of data analysis.

### 2.9. Sample Size and Power Analysis

Sample size was calculated based on the primary outcome of the study, the clinical pregnancy rate. Previous studies evaluating the effect of hyaluronan-rich transfer media on clinical pregnancy have reported clinical pregnancy rates approximately 9% higher in EmbryoGlue groups compared with control groups [9,10]. Based on these data, the clinical pregnancy rate was anticipated to be 31% in the control group and 44% in the EmbryoGlue group (an absolute increase of 13%). Assuming a two-sided significance level of 5% (α = 0.05) and 80% power (1 − β = 0.80), a minimum of 220 patients per group, and 440 patients in total, was calculated to be required to compare two independent proportions. The power analysis was performed using G*Power software (version 3.1.9.7; Heinrich Heine University, Düsseldorf, Germany) [11]. A total of 515 patients meeting the eligibility criteria (EmbryoGlue group, *n* = 232; control group, *n* = 283) were enrolled during the study period, exceeding the calculated minimum sample size (*n* = 440).

### 2.10. Statistical Analysis

The distribution of continuous variables was assessed using the Kolmogorov–Smirnov test and histogram/Q–Q plots. Normally distributed variables were presented as mean ± standard deviation and compared using the independent-samples *t*-test; non-normally distributed variables were presented as median (interquartile range) and compared using the Mann–Whitney U test. Categorical variables were presented as number (percentage) and compared using the Pearson chi-square test or, where appropriate, Fisher’s exact test.

Multivariable logistic regression analysis was performed to identify independent determinants of clinical pregnancy and live birth. Variables known from the literature to be associated with clinical pregnancy and/or showing significance at *p* < 0.10 in univariate analyses were entered into the model: maternal age, AMH level, endometrial thickness, embryo quality (presence of a good-quality blastocyst), fibroid size (continuous variable, cm), presence of ≥2 fibroids, and EmbryoGlue use. An EmbryoGlue × fibroid-size interaction term was also added to the model to assess whether the effect of EmbryoGlue was modified by fibroid size. Results were reported as adjusted odds ratios (aOR) with 95% confidence intervals (CI). To facilitate interpretation of the EmbryoGlue main effect at a clinically representative fibroid size rather than at a fibroid size of zero, fibroid size was mean-centered at 3 cm (the approximate sample mean) prior to construction of the EmbryoGlue × fibroid-size interaction term.

Subgroup analyses according to fibroid size were considered exploratory (hypothesis-generating) in nature, owing to the limited sample sizes of the subgroups and the absence of correction for multiple comparisons.

A two-sided *p* value <0.05 was considered statistically significant in all analyses. Analyses were performed using IBM SPSS Statistics for Windows, version 26.0 (IBM Corp., Armonk, NY, USA).

## 3. Results

### 3.1. Baseline Characteristics

**Figure 1:**
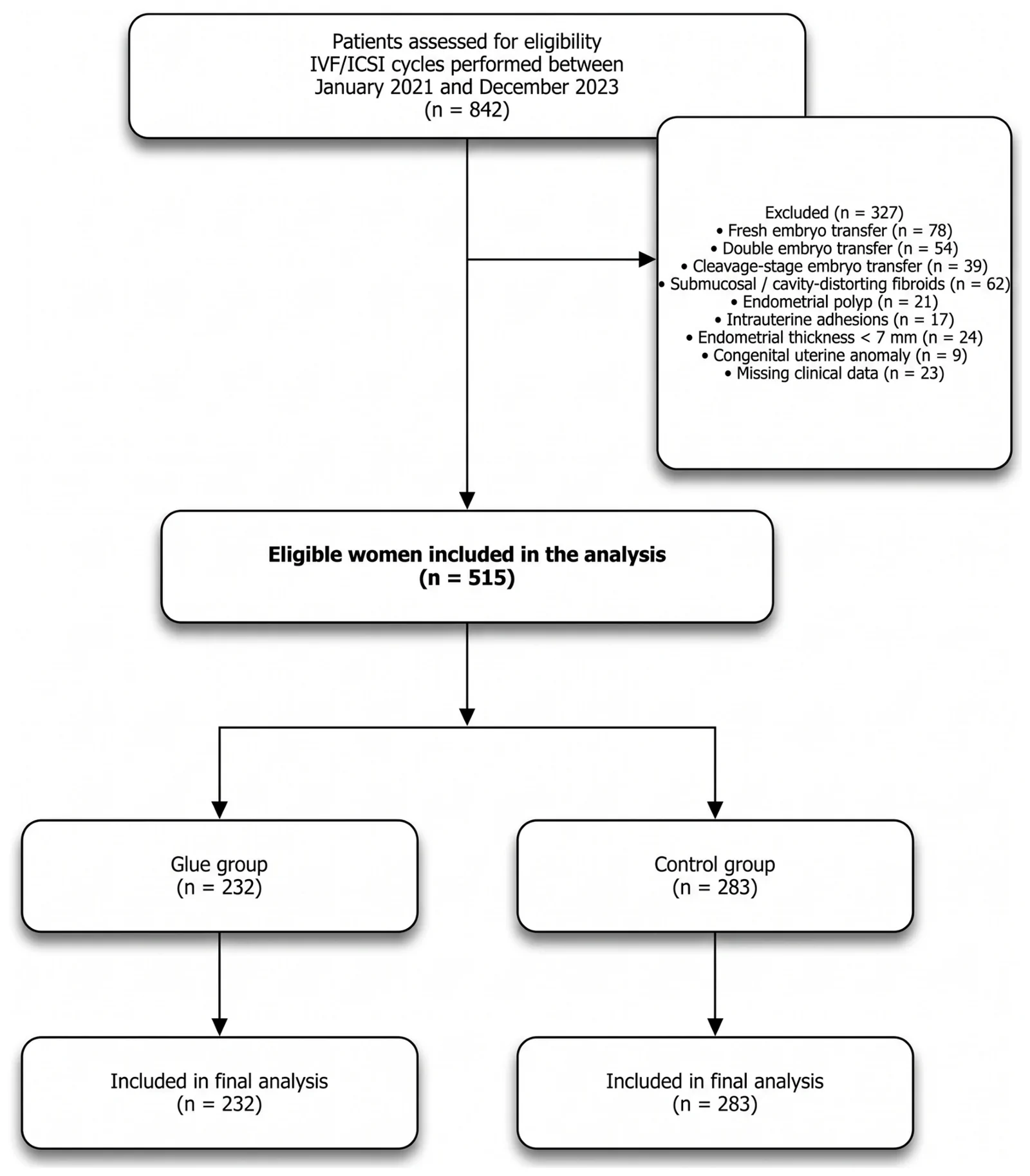
Flow diagram of patient selection according to the STROBE statement.

A total of 232 women in the EmbryoGlue group and 283 women in the control group were included in the analysis (Figure 1).

Baseline characteristics showed some between-group imbalances, including a significantly higher maternal age in the EmbryoGlue group (34.4 ± 3.2 vs. 33.5 ± 3.4 years; *p* = 0.002), as well as a non-significantly greater endometrial thickness and proportion of good-quality blastocysts in the control group (Table 1).

The distribution of infertility etiologies was similar between groups, with unexplained infertility representing the most common category in both the EmbryoGlue (44.8%) and control (48.1%) groups, followed by tubal factor, male factor, and endometriosis; none of these categories differed significantly between groups (Table 2).

### 3.2. Primary Reproductive Outcomes

The EmbryoGlue group had significantly higher rates of positive pregnancy test, clinical pregnancy, and live birth compared with the control group, while early pregnancy loss rates among women who achieved a clinical pregnancy were similar between groups (Table 3).

### 3.3. Subgroup Analysis According to Fibroid Size

When outcomes were stratified by intramural fibroid size, the magnitude of the between-group differences varied across fibroid-size categories, with the largest differences observed in women with fibroids >4 cm (Table 4).

The interaction between EmbryoGlue use and fibroid size was statistically significant for both clinical pregnancy (*p* for interaction = 0.011) and live birth (*p* for interaction = 0.028) (Table 5 and Table 6).

In exploratory subgroup analyses, clinical pregnancy rates did not differ significantly between groups among women with fibroids <2 cm (38.5% vs. 25.8%; *p* = 0.060) or 2–4 cm (38.2% vs. 27.7%; *p* = 0.124). Among women with fibroids >4 cm, clinical pregnancy rates were significantly higher in the EmbryoGlue group (46.2% vs. 27.4%; *p* = 0.029). Similar patterns were observed for live birth, with rates of 29.5% versus 18.3% in the <2 cm group (*p* = 0.067), 28.1% versus 20.8% in the 2–4 cm group (*p* = 0.241), and 33.8% versus 17.7% in the >4 cm group (*p* = 0.039) (Table 4).

These subgroup comparisons were unadjusted and were considered exploratory. Importantly, the statistical significance of an association within an individual subgroup does not by itself establish that the treatment effect differs significantly between subgroups; this question was assessed using the prespecified EmbryoGlue × fibroid-size interaction term in the multivariable model.

### 3.4. Multivariable Analysis

In the multivariable logistic regression model for clinical pregnancy, at the reference fibroid size of 3 cm, EmbryoGlue use was independently associated with higher odds of clinical pregnancy, and a significant interaction between EmbryoGlue use and intramural fibroid size was observed, indicating that this association varied according to fibroid size (Table 5).

Advanced maternal age and increasing fibroid size were independently associated with lower odds of clinical pregnancy, whereas higher AMH, greater endometrial thickness, and transfer of a good-quality embryo were independently associated with higher odds; the presence of two or more fibroids was not an independent predictor (Table 5). Therefore, the main effect of EmbryoGlue should be interpreted in the context of fibroid size rather than as a uniform treatment effect across all patients.

In the multivariable logistic regression model for live birth, at the reference fibroid size of 3 cm, EmbryoGlue use was not independently associated with live birth as a uniform main effect, whereas the EmbryoGlue × fibroid-size interaction remained significant, indicating that this association varied according to fibroid size (Table 6).

Maternal age and increasing fibroid size were associated with lower odds of live birth, whereas higher AMH, greater endometrial thickness, and transfer of a good-quality embryo were associated with higher odds (Table 6).

To facilitate clinical interpretation of the EmbryoGlue × fibroid size interaction terms derived from the multivariable models (Table 5 and Table 6), adjusted predicted probabilities were computed across fibroid diameters from 2 cm to 6 cm (Table 7). While predicted clinical pregnancy and live birth rates declined progressively with increasing fibroid diameter in the control group, they increased in the EmbryoGlue group. Accordingly, the absolute benefit associated with EmbryoGlue expanded substantially as fibroid diameter increased, reaching an absolute difference of +50.1 percentage points for clinical pregnancy (Panel A) and +29.1 percentage points for live birth (Panel B) at 6 cm (Table 7).

To further evaluate the robustness of these findings to residual confounding, sensitivity analyses were performed in which the multivariable models were additionally adjusted for previous failed embryo transfer, treatment year, infertility duration, and blastocyst developmental day (Day-6 vs. Day-5) (Table 8). After this additional adjustment, EmbryoGlue use at the reference fibroid size of 3 cm remained independently associated with clinical pregnancy (aOR 1.82, 95% CI 1.09–3.04; *p* = 0.022), and the EmbryoGlue × fibroid-size interaction remained statistically significant for both clinical pregnancy (aOR 1.58, 95% CI 1.05–2.38; *p* = 0.028) and live birth (aOR 1.54, 95% CI 1.01–2.35; *p* = 0.045). Treatment year was independently associated with both outcomes (*p* = 0.018 and *p* = 0.012, respectively), while previous failed embryo transfer, infertility duration, and blastocyst developmental day were not independent predictors of either outcome. These findings indicate that the observed associations were not substantially explained by these additional potential confounders.

As a sensitivity analysis, the primary multivariable models were additionally adjusted for previous failed embryo transfer, treatment year, infertility duration, and blastocyst developmental day (Day-6 vs. Day-5); results are presented in Table 8.

## 4. Discussion

In this retrospective cohort study, EmbryoGlue use was associated with significantly higher rates of positive pregnancy, clinical pregnancy, and live birth in women with non-cavity-distorting intramural fibroids undergoing frozen–thawed blastocyst transfer, while early pregnancy loss rates were comparable between groups. In multivariable analysis, at the reference fibroid size of 3 cm, EmbryoGlue use was independently associated with higher odds of clinical pregnancy, after adjustment for relevant clinical and embryological factors; advanced maternal age and increasing fibroid size were independently associated with lower odds of clinical pregnancy, whereas higher AMH, greater endometrial thickness, and good-quality blastocyst transfer were associated with improved outcomes. Importantly, a significant EmbryoGlue × fibroid-size interaction indicated that this association varied by fibroid size rather than representing a uniform effect across the cohort. A separate model for live birth showed a similar conditional pattern (adjusted OR for interaction 1.68; 95% CI 1.06–2.67; *p* = 0.028).

### 4.1. Strengths and Limitations

This study benefits from several design features worth highlighting: it draws on a sizeable cohort specifically focused on non-cavity-distorting intramural fibroids, applies a single and consistent transfer protocol across all patients, and captures a detailed set of variables known to bear on IVF success—including age, BMI, infertility duration, hormonal profile, AFC, endometrial thickness, and embryo quality—which made it possible to adjust statistically for the major confounders.

Several limitations warrant discussion, however. Because the design was retrospective and non-randomized, with treatment chosen by patients rather than assigned at random, both selection bias and residual confounding remain possible. Even though EmbryoGlue was offered as a standard option to every eligible patient irrespective of prognosis, embryo quality, ovarian reserve, or fibroid size, the control group happened to have a somewhat thicker endometrium, a numerically greater share of good-quality blastocysts, and a marginally better ovarian reserve profile—imbalances that, if anything, would work against rather than inflate any apparent benefit of EmbryoGlue.

Testing multiple subgroups and outcomes without a formal correction for multiplicity (such as a Bonferroni adjustment) raises the likelihood of a chance false-positive finding, so the subgroup-level results are best treated as exploratory rather than confirmatory. The sensitivity analyses that incorporated additional covariates (Table 8) produced largely consistent results, though adding these parameters brought the ratio of events to predictors—particularly for live birth—close to the commonly recommended minimum, meaning that some degree of residual confounding cannot be entirely ruled out.

We did not systematically document the precise location of each fibroid, total fibroid volume, or uterine peristalsis. While the presence of two or more fibroids was recorded and did not differ meaningfully between the groups (43.1% vs. 36.7%; *p* = 0.14), we lacked systematic data on the exact fibroid count or overall fibroid burden, relying instead on the diameter of the single largest fibroid for size classification. As a result, two patients sharing the same maximum fibroid diameter but carrying a different overall fibroid burden would have been treated identically in our analysis, and our findings are further limited to frozen-thawed transfer cycles. How a fibroid sits in relation to the endometrial and serosal surfaces—captured by its FIGO type—may shape reproductive outcomes through pathways distinct from size alone. The distribution of FIGO type 3, 4, and 5 fibroids was similar across the two groups (*p* = 0.969), but our sample was not large enough to detect outcome differences tied to specific FIGO types or to test for an EmbryoGlue × FIGO-type interaction, a question future studies will need to address.

### 4.2. Outcome Baseline

Whether hyaluronic acid-enriched transfer media genuinely benefit assisted reproduction has been debated for some time. A number of systematic reviews—including one from the Cochrane group—conclude that high-molecular-weight hyaluronic acid preparations can produce a modest uptick in implantation and live birth rates, though the effect is far from consistent across different patient populations [10,12,13,14]. Among individual studies, Bontekoe and colleagues found a small but statistically significant improvement in live birth rate with adhesion-enhancing compounds [7], Singh and colleagues reported better pregnancy rates in general fresh IVF cycles [15], and Atkinson and Woodland proposed that the benefit may be concentrated in subgroups facing particular implantation difficulties [16].

Very little published work has looked specifically at EmbryoGlue in women with non-cavity-distorting intramural fibroids, even though these fibroids are known to lower clinical pregnancy and live birth rates on their own, without any cavity distortion [17]. Set against this background, the relative gain in clinical pregnancy and live birth we observed was considerably larger than the modest benefit typically reported in unselected IVF populations [7]. Specifically, among women with fibroids exceeding 4 cm, clinical pregnancy (46.2% vs. 27.4%) and live birth (33.8% vs. 17.7%) rates were both markedly higher in the EmbryoGlue group—a difference substantial enough to be clinically meaningful, and one that fits with the idea that hyaluronan-enriched media may offer proportionally greater benefit precisely where implantation is already compromised by anatomical or functional uterine alterations [18].

### 4.3. Interpretation of Key Findings

The multivariable model reproduced several well-established predictors of IVF success. Each additional year of maternal age lowered the odds of clinical pregnancy (aOR 0.94; 95% CI 0.90–0.98; *p* = 0.003), whereas higher AMH levels (aOR 1.15; 95% CI 1.02–1.30; *p* = 0.021), a thicker endometrium (aOR 1.17; 95% CI 1.04–1.32; *p* = 0.009), and transfer of a good-quality blastocyst (aOR 1.58; 95% CI 1.04–2.40; *p* = 0.032) each raised it. These directions of effect align with the recognized contributions of endometrial receptivity and embryo quality to implantation [19,20]. Overall, the model fit the data well (χ^2^ = 34.7; *p* < 0.001; Nagelkerke R^2^ = 0.29).

Taking a fibroid size of 3 cm as the point of reference, EmbryoGlue use remained independently linked to higher odds of clinical pregnancy (aOR 2.05; 95% CI 1.20–3.50; *p* = 0.008), and the interaction between EmbryoGlue use and fibroid size continued to reach statistical significance (aOR 1.74; 95% CI 1.13–2.68; *p* = 0.011). Taken together, these results point to an association that shifts with fibroid size rather than one that applies uniformly across the cohort. This should not be read as evidence of a fixed treatment cutoff at 4 cm; rather, the interaction term reflects a gradual shift in the strength of the EmbryoGlue–outcome relationship as fibroid size increases. Table 7 presents the corresponding adjusted predicted probabilities across a clinically relevant range of fibroid sizes.

Taken as a whole, our data point to an EmbryoGlue–outcome relationship that is not constant but shifts with fibroid size, becoming most pronounced among women with fibroids exceeding 4 cm; nonetheless, this study was not designed to identify a precise size cutoff for guiding treatment decisions.

Some baseline imbalances were present. The EmbryoGlue group had a significantly higher maternal age (34.4 ± 3.2 vs. 33.5 ± 3.4 years; *p* = 0.002), which would be expected to work against rather than inflate any apparent EmbryoGlue benefit, given that advanced maternal age was independently associated with lower odds of both outcomes in the adjusted models. In addition, the control group had a thicker endometrium (9.2 ± 1.4 vs. 8.8 ± 1.3 mm; *p* = 0.07), a numerically higher proportion of good-quality blastocysts (69.6% vs. 61.6%; *p* = 0.051), and a marginally more favorable ovarian reserve profile. Collectively, these baseline differences would, if anything, be expected to bias the unadjusted comparisons against rather than in favor of EmbryoGlue. Yet clinical pregnancy (40.5% vs. 26.9%) and live birth (30.2% vs. 19.1%) rates both favored the EmbryoGlue group, and the main effect at the reference fibroid size together with the persistent interaction argue against baseline imbalance as the sole explanation. Residual confounding nonetheless cannot be ruled out, given that treatment was allocated according to patient preference rather than by randomization. Stratified analyses pointed in the same direction, albeit without a strictly linear progression. Numerically higher clinical pregnancy and live birth rates favored the EmbryoGlue group in every fibroid-size stratum, yet the between-group gap reached statistical significance only in the >4 cm stratum (clinical pregnancy 27.4%→46.2%, *p* = 0.029; live birth 17.7%→33.8%, *p* = 0.039), not in the <2 cm (*p* = 0.060) or 2–4 cm (*p* = 0.124) strata. The comparatively modest gap in the middle category may simply reflect the smaller sample within that stratum; given the lack of correction for multiple testing, these stratified results should be viewed as exploratory. Even so, the continuous-fibroid-size model—relying on a single interaction test rather than repeated subgroup comparisons—pointed to a significant, though non-linear, shift in the EmbryoGlue–outcome relationship across the fibroid-size spectrum (*p* for interaction = 0.011), a pattern broadly consistent with mechanistic work suggesting that intramural fibroids can compromise uterine contractility, local blood flow, and endometrial receptivity [16,20].

Early pregnancy loss occurred at comparable rates in both groups (25.5% vs. 28.9%; *p* = 0.620), suggesting that any benefit of EmbryoGlue is exerted mainly at the point of implantation and early pregnancy establishment, rather than by lowering the risk of miscarriage once a pregnancy is already underway—consistent with the broader evidence that early losses are chiefly driven by embryonic chromosomal abnormalities and maternal factors [21,22].

### 4.4. Clinical Implications

Whether to proceed with myomectomy in women with non-cavity-distorting intramural fibroids remains a difficult clinical call, weighed against risks such as bleeding, adhesion formation, uterine scarring, and diminished ovarian reserve, and complicated further by uncertainty over how much these fibroids actually affect IVF success [1,2,3,20]. Our data suggest that a hyaluronan-enriched transfer medium could be worth exploring further as a non-surgical adjunct in this patient group.

That said, EmbryoGlue is not a substitute for surgical management when fibroids distort the cavity, produce marked symptoms, or raise concern for malignancy. These findings are hypothesis-generating and should not be interpreted as supporting the routine use of EmbryoGlue in patients with non-cavity-distorting fibroids, including those ≥4 cm. Although the EmbryoGlue × fibroid-size interaction was statistically significant, the subgroup findings did not demonstrate a strictly linear pattern across fibroid-size categories, and the largest between-group differences observed in women with fibroids >4 cm require confirmation in prospective controlled studies before any change in clinical practice can be recommended [23,24,25].

### 4.5. Future Research Questions

Several questions warrant prospective investigation: whether the fibroid-size-dependent benefit is genuine or reflects residual confounding; the biological mechanism underlying this fibroid-size-dependent association (uterine contractility, perfusion, or receptivity).

## 5. Conclusions

In summary, women with non-cavity-distorting intramural fibroids who received EmbryoGlue during frozen–thawed single blastocyst transfer showed higher rates of positive pregnancy test, clinical pregnancy, and live birth than those who did not. At the reference fibroid size of 3 cm, EmbryoGlue use was independently linked to clinical pregnancy in the multivariable model, and its interaction with fibroid size indicates that this association is not uniform but instead depends on fibroid size—a pattern also reflected in the exploratory subgroup analyses. Given the retrospective, non-randomized design, these findings are best regarded as hypothesis-generating rather than as grounds for changing current practice. Confirmation through prospective randomized trials will be necessary, both to validate these results and to clarify which patients stand to benefit most, before EmbryoGlue can be recommended as a routine adjunct in this population.

## Figures and Tables

**Table 1:** Baseline demographic and reproductive characteristics of the study groups.

Variable	EmbryoGlue Group (*n* = 232)	Control Group (*n* = 283)	*p*-Value
FIGO type 3, *n* (%)	82 (35.3)	103 (36.4)	
FIGO type 4, *n* (%)	102 (44.0)	122 (43.1)	
FIGO type 5, *n* (%)	48 (20.7)	58 (20.5)	0.969
Age (years) ^a^	34.4 ± 3.2	33.5 ± 3.4	0.002
BMI (kg/m^2^)	24.9 ± 3.5	25.3 ± 3.4	0.40
Duration of infertility (years)	5.8 ± 3.4	4.9 ± 3.1	0.07
Basal FSH (IU/L)	8.2 ± 2.3	7.8 ± 2.1	0.19
AMH (ng/mL)	2.18 ± 1.35	2.55 ± 1.48	0.060
AFC (*n*)	10.8 ± 4.4	12.0 ± 4.7	0.058
Intramural fibroid size (cm)	3.0 ± 1.2	2.8 ± 1.1	0.21
≥2 fibroids, *n* (%) ^b^	100 (43.1)	104 (36.7)	0.14
Previous failed ET ≥1, *n* (%) ^b^	92 (39.7)	103 (36.4)	0.448
Endometrial thickness (mm)	8.8 ± 1.3	9.2 ± 1.4	0.07
Day-5 blastocyst transfer, *n* (%) ^b^	161 (69.4)	216 (76.3)	0.077
Day-6 blastocyst transfer, *n* (%) ^b^	71 (30.6)	67 (23.7)	0.077
Good-quality blastocyst (AA/AB/BA), *n* (%) ^b^	143 (61.6)	197 (69.6)	0.051
Treatment year, *n* (%)			<0.001
2021	25 (10.8)	65 (23.0)	
2022	35 (15.1)	60 (21.2)	
2023	45 (19.4)	58 (20.5)	
2024	57 (24.6)	52 (18.4)	
2025	70 (30.2)	48 (17.0)	

Data are presented as mean ± standard deviation or number (%). BMI, body mass index; FSH, follicle-stimulating hormone; AMH, anti-Müllerian hormone; AFC, antral follicle count; ET, embryo transfer; FIGO, International Federation of Gynecology and Obstetrics. FIGO type distribution was compared using the 3 × 2 Pearson chi-square test. All continuous variables not marked a were compared using the Mann–Whitney U test. ^a^ Independent-samples *t*-test. ^b^ Pearson chi-square test.

**Table 2:** Causes of infertility in the study groups.

Variable	EmbryoGlue (*n* = 232)	Control (*n* = 283)	*p*-Value
Unexplained infertility	104 (44.8)	136 (48.1)	0.46
Tubal factor	44 (19.0)	58 (20.5)	0.67
Male factor	50 (21.6)	53 (18.7)	0.42
Endometriosis	34 (14.7)	36 (12.7)	0.52

Data are presented as number (%). All variables were compared using the Pearson chi-square test.

**Table 3:** Reproductive outcomes of the study groups.

Variable	EmbryoGlue Group (*n* = 232)	Control Group (*n* = 283)	*p*-Value
Positive pregnancy test, *n* (%)	116 (50.0)	93 (32.9)	<0.001
Clinical pregnancy, *n* (%)	94 (40.5)	76 (26.9)	0.001
Live birth, *n* (%)	70 (30.2)	54 (19.1)	0.003
Early pregnancy loss ^a^, *n* (%)	24/94 (25.5)	22/76 (28.9)	0.620

Data are presented as number (%). All variables were compared using the Pearson chi-square test. ^a^ Calculated among women with a clinical pregnancy.

**Table 4:** Reproductive outcomes according to intramural fibroid size.

Fibroid Size	Group	Clinical Pregnancy, *n* (%)	*p*-Value	Live Birth, *n* (%)	*p*-Value
<2 cm	EmbryoGlue (*n* = 78)	30 (38.5)	0.060	23 (29.5)	0.067
	Control (*n* = 120)	31 (25.8)		22 (18.3)	
2–4 cm	EmbryoGlue (*n* = 89)	34 (38.2)	0.124	25 (28.1)	0.241
	Control (*n* = 101)	28 (27.7)		21 (20.8)	
>4 cm	EmbryoGlue (*n* = 65)	30 (46.2)	0.029	22 (33.8)	0.039
	Control (*n* = 62)	17 (27.4)		11 (17.7)	

Data are presented as number (%). Between-group comparisons within each fibroid-size stratum were performed using the Pearson chi-square test. Formal assessment of effect modification was based on the EmbryoGlue × fibroid-size interaction term in the multivariable logistic regression models (Table 5 and Table 6).

**Table 5:** Multivariable logistic regression analysis for clinical pregnancy.

Variable	Adjusted OR	95% CI	*p*-Value
EmbryoGlue use (at 3 cm fibroid size)	2.05	1.20–3.50	0.008
Maternal age (per year)	0.94	0.90–0.98	0.003
AMH (per ng/mL)	1.15	1.02–1.30	0.021
Endometrial thickness (per mm)	1.17	1.04–1.32	0.009
Good-quality embryo (AA/AB/BA)	1.58	1.04–2.40	0.032
Intramural fibroid size (per cm)	0.77	0.63–0.94	0.026
≥2 fibroids	0.92	0.61–1.39	0.690
EmbryoGlue × (fibroid size − 3 cm)	1.74	1.13–2.68	0.011

Odds ratios were adjusted simultaneously for all variables listed. OR, odds ratio; CI, confidence interval; AMH, anti-Müllerian hormone. Model χ^2^ = 34.7, *p* < 0.001; Nagelkerke R^2^ = 0.29.

**Table 6:** Multivariable logistic regression analysis for live birth.

Variable	Adjusted OR	95% CI	*p*-Value
EmbryoGlue use (at 3 cm fibroid size)	1.35	0.79–2.31	0.274
Maternal age (per year)	0.92	0.87–0.97	0.002
AMH (per ng/mL)	1.13	1.00–1.29	0.049
Endometrial thickness (per mm)	1.15	1.01–1.31	0.036
Good-quality embryo (AA/AB/BA)	1.72	1.06–2.80	0.028
Intramural fibroid size (per cm)	0.74	0.59–0.93	0.010
≥2 fibroids	0.88	0.56–1.39	0.586
EmbryoGlue × fibroid size	1.68	1.06–2.67	0.028

Odds ratios were adjusted simultaneously for all variables listed. OR, odds ratio; CI, confidence interval; AMH, anti-Müllerian hormone. Model χ^2^ = 31.9, *p* < 0.001; Nagelkerke R^2^ = 0.27.

**Table 7:** (Panel A). Adjusted predicted probabilities of clinical pregnancy according to fibroid size and transfer medium. (Panel B). Adjusted predicted probabilities of live birth according to fibroid size and transfer medium.

**Panel A**			
**Fibroid Size**	**Control**	**EmbryoGlue**	**Absolute Difference**
2 cm	32.4%	36.1%	+3.7 percentage points
3 cm	27.0%	43.1%	+16.1 percentage points
4 cm	22.2%	50.4%	+28.2 percentage points
5 cm	18.0%	57.6%	+39.6 percentage points
6 cm	14.4%	64.6%	+50.1 percentage points
**Panel B**			
**Fibroid Size**	**Control**	**EmbryoGlue**	**Absolute Difference**
2 cm	24.1%	20.3%	−3.8 percentage points
3 cm	19.0%	24.1%	+5.1 percentage points
4 cm	14.8%	28.2%	+13.5 percentage points
5 cm	11.4%	32.9%	+21.5 percentage points
6 cm	8.7%	37.8%	+29.1 percentage points

(Panel A): Predicted probabilities were derived from the multivariable logistic regression model for clinical pregnancy (Table 5), with fibroid size centered at 3 cm and all other covariates held at their reference values. These model-based estimates are presented to facilitate clinical interpretation of the EmbryoGlue × fibroid-size interaction and should not be interpreted as evidence of a causal treatment effect, given the observational and exploratory nature of the analysis. (Panel B): Predicted probabilities were derived from the multivariable logistic regression model for live birth (Table 6), with fibroid size centered at 3 cm and all other covariates held at their reference values. These model-based estimates are presented to facilitate clinical interpretation of the EmbryoGlue × fibroid-size interaction and should not be interpreted as evidence of a causal treatment effect, given the observational and exploratory nature of the analysis. At smaller fibroid sizes, the model-based estimates show a modest crossover, likely reflecting extrapolation of a linear interaction term rather than a true adverse association.

**Table 8:** Sensitivity analyses with additional adjustment for potential residual confounders.

Variable	Clinical Pregnancy aOR (95% CI)	*p*-Value	Live Birth aOR (95% CI)	*p*-Value
EmbryoGlue use (at 3 cm fibroid size)	1.82 (1.09–3.04)	0.022	1.24 (0.73–2.11)	0.426
Maternal age (per year)	0.94 (0.90–0.98)	0.005	0.93 (0.88–0.98)	0.006
AMH (per ng/mL)	1.13 (1.00–1.28)	0.047	1.11 (0.98–1.27)	0.099
Endometrial thickness (per mm)	1.15 (1.02–1.30)	0.023	1.13 (0.99–1.29)	0.068
Good-quality embryo	1.49 (1.01–2.21)	0.046	1.61 (1.01–2.58)	0.046
Fibroid size (per cm)	0.79 (0.65–0.96)	0.019	0.77 (0.61–0.97)	0.026
≥2 fibroids	0.93 (0.61–1.42)	0.739	0.90 (0.56–1.44)	0.657
Previous failed embryo transfer	0.76 (0.54–1.07)	0.117	0.72 (0.50–1.04)	0.080
Treatment year	1.12 (1.02–1.23)	0.018	1.14 (1.03–1.26)	0.012
Infertility duration (per year)	0.97 (0.92–1.03)	0.316	0.96 (0.90–1.02)	0.186
Day-6 vs. Day-5 blastocyst	0.83 (0.59–1.17)	0.288	0.80 (0.55–1.16)	0.239
EmbryoGlue × fibroid size	1.58 (1.05–2.38)	0.028	1.54 (1.01–2.35)	0.045

Multivariable logistic regression models for clinical pregnancy and live birth, additionally adjusted for previous failed embryo transfer, treatment year, infertility duration, and blastocyst developmental day (Day-6 vs. Day-5), beyond the covariates included in the primary models (Table 5 and Table 6). The EmbryoGlue main effect and the EmbryoGlue × fibroid-size interaction remained directionally consistent with the primary analyses after this additional adjustment. aOR, adjusted odds ratio; CI, confidence interval; AMH, anti-Müllerian hormone.

## Data Availability

The datasets generated and/or analyzed during the current study are not publicly available due to institutional data protection policies but are available from the corresponding author on reasonable request, subject to institutional and ethical approval.

## Abbreviations

The following abbreviations are used in this manuscript:

AFC	Antral follicle count
AMH	Anti-Müllerian hormone
aOR	Adjusted odds ratio
ART	Assisted reproductive technology
CI	Confidence interval
FIGO	International Federation of Gynecology and Obstetrics
FSH	Follicle-stimulating hormone
HRT	Hormone replacement therapy
ICSI	Intracytoplasmic sperm injection
IVF	In vitro fertilization
SIS	Saline infusion sonography
STROBE	Strengthening the Reporting of Observational Studies in Epidemiology
TVUS	Transvaginal ultrasonography
